# The genomic features associated with high-risk multiple myeloma

**DOI:** 10.18632/oncotarget.26269

**Published:** 2018-10-26

**Authors:** Brian A. Walker, Gareth J. Morgan

**Affiliations:** Myeloma Institute, University of Arkansas for Medical Sciences, Little Rock, AR

**Keywords:** multiple myeloma, high-risk, Double-Hit, risk stratification, personalised treatment

Multiple myeloma (MM) is a malignancy of plasma cells for which the outcome of treatment has improved over the last decade. Yet, despite this overall improvement a substantial proportion of patients have not benefited as much as would be desired. A solution to improve the outcome for these patients is to segment the disease into discrete biological entities and to direct clinical trial efforts specifically to these subgroups with the aim of optimizing therapeutic strategies. Advances in genomic studies provide an important tool to segment MM into individual risk strata based on the idea that acquired genetic events drive both the biology and risk status of individual cases.

A key subgroup worthy of specific attention is high-risk myeloma, which has a particularly poor outcome. This group constitutes a significant proportion of newly diagnosed MM (NDMM) cases who do not seem to have benefited as much as other groups from recent therapeutic advances, with treatment resistance and early relapse being common. Identifying these patients at presentation, when their therapy can be modified from the “one size fits all” approach, could result in them being included in clinical trials designed to address their poor prognosis. This is becoming increasingly important as a number of new therapeutic strategies have recently become available, including chimeric antigen receptor (CAR)-T cell therapies and bi-specific antibodies, which while they are currently being evaluated for relapse refractory disease could be effective for this high risk NDMM segment [1].

MM can effectively risk stratified by only a limited number of key genomic abnormalities with cases carrying the etiologic structural variants including t(4;14), t(14;16), and t(14;20) being associated with a poor prognosis [2]. Select secondary events are also associated with poor prognosis including del1p, amp1q, and del17p. To improve simple risk stratifications some of these molecular abnormalities have been incorporated into the International Staging System (ISS), that is based on albumin and β_2_m, to create the revised-ISS (R-ISS) [3, 4]. The R-ISS is able to separate patients into three groups with different median overall survival (OS) rates (5-year OS rate = 82% for R-ISS I, 62% for R-ISS II, and 40% for R-ISS III).

The advent of next generation sequencing technologies has exponentially increased the volume of genetic data available and the work done on malignant plasma cells has shown that acquired genetic events are key components driving clinical risk status. Consequently, it follows that the incorporation of all prognostically relevant genetic data into a clinical risk stratification system could substantially improve its sensitivity and specificity.

As part of an international collaboration, the myeloma genome project, we have used a combination of whole genome, exome, and RNA-sequencing to analyze the largest dataset of newly diagnosed MM trial patients assembled to date. We analyzed these cases to identify “genetic drivers” that adversely impact prognosis in a genome-wide unbiased manner [5, 6]. Using 1273 NDMM patient samples we determined primary events of translocations and hyperdiploidy, as well as copy number abnormalities and mutations.

Analysis of the translocation groups, combined with copy number abnormalities and mutational data revealed a series of interesting oncogenic dependencies, where the initial events predispose the tumors to specific fates. For example, tumors with a t(4;14) are associated with mutations in *FGFR3*, *PRKD2*, and *DIS3*, whereas those with a t(11;14) are associated with mutations in *CCND1*, *IRF4*, and *LTB*. The t(14;16) group is associated with a higher mutational burden that is enriched for mutations with a signature that implies they are generated by aberrant APOBEC cytidine deaminase activity. *BRAF* mutations in MM are more common at codon V600, but in the t(14;16) group this is not the case with the D594 mutation being predominant, and this may be a function of the aberrant APOBEC activity. Tumors with hyperdiploidy are associated with mutations in *FAM46C*, secondary translocations involving the *MYC* locus at 8q24, and trisomy of chromosome 11.

A univariate and multivariate analysis of these genomic factors was carried out to determine the association of these genetic drivers with outcome. At this stage, key clinical and biochemical parameters were added, including ISS and age. The multivariate analysis identified biallelic inactivation of *TP53*, gain or amplification of 1q, ISS II or III, and age >65 years as being associated with poor progression free survival (PFS) and OS, as well as a loss of heterozygosity score of >4.6% which was associated with PFS only. To develop a risk classification system applicable to individual patients we performed recursive-partitioning analysis of the key parameters associated with PFS. This analysis identified seven terminal nodes that were combined to yield three groups, defining low, intermediate and high-risk cases. Importantly the high-risk group accounted for approximately 6% of NDMM patients and was comprised of patients with either biallelic *TP53* alterations, or amplification of 1q21 and ISS III. As such, we designated this high-risk group as Double-Hit myeloma.

The Double-Hit group has an extremely poor outcome with a median PFS of only 15.4 months and OS of 20.7 months. Importantly, we were able to validate the size and adverse outcome associated with this group in an independent dataset. The outcome of this group is similar to what is seen in relapsed refractory MM, a subset of cases that have formed the basis of our drug development strategies for the last 20 years. Thus, clinical trials could be both ethically and rationally designed to address the poor prognosis of this group in the newly diagnosed setting. Taking this approach would prevent the long delays involved in developing new strategies for relapse refractory patients and then moving them forward.

These patients are easy to identify as the test relies on biochemical markers for ISS, which are routinely performed, and only two genetic markers: *TP53* and amp1q. To date mutational status of *TP53* is not routinely performed in MM, yet our studies indicate that both mutation and deletion of *TP53* are extremely relevant and the identification of biallelic *TP53* inactivation should be a clinical priority. The role of 1q21 gain has also been clarified with the adverse prognosis associated with a short PFS being driven by the 3% of cases with amplification (>3 copies) in the context of ISS III. The genomic technologies to detect such abnormalities are in widespread use and give results in a clinically relevant timeline. Further, such technologies give a binary answer either showing the presence or absence of the lesion and do not rely on the definition of arbitrary thresholds such as is required for cytogenetic technologies.

The “Double Hit” group does not replace previous risk markers identified by iFISH but rather it identifies a distinct subgroup of patients at particularly high-risk of early progression and death that are suitable for entry into trials of novel therapies aimed at improving their outcome. Given the frequency of other mutational events in NDMM it is unlikely that, given our current knowledge of the impact and frequency of mutations, that the size of the group will increase substantially unless other driver mechanisms are identified. In this context we clearly show that despite the size of the study that we are missing genetic drivers in a substantial proportion of cases. Such mechanisms may be currently unknown or occur in portions of the genome we have not studied.
